# How variable are the volumetric measurements from gated perfusion SPECT when a one-day stress-rest protocol is used?

**DOI:** 10.1007/s12350-018-1253-4

**Published:** 2018-03-15

**Authors:** C. Fielder Camm, Alexander Emery, Elizabeth Rose-Innes, Sergei Pavlitchouk, Nikant Sabharwal, Andrew D. Kelion

**Affiliations:** 1 Radcliffe Department of Medicine, University of Oxford, Oxford, UK; 2 Department of Cardiology, Oxford University Hospitals NHS Foundation Trust, Oxford, UK; 3 Keble College, University of Oxford, Oxford, UK

**Keywords:** Nuclear cardiology, SPECT, ischaemic heart disease, reproducibility, technetium-99m

## Abstract

**Background:**

Using myocardial perfusion scintigraphy (MPS), an increase in left ventricular (LV) volumes or a decrease in ejection fraction (EF) from rest to stress may be clinically important. The variation in these measures between the low-dose stress acquisition and high-dose rest acquisition in a one-day stress-rest protocol has not been established. We assessed the reproducibility of gated volumetric indices between stress and rest and the normal variation in ungated TID ratio for a one-day stress-rest ^99m^Tc-tetrofosmin protocol.

**Methods:**

Two thousand and one hundred and fifty eight (2158) ^99m^Tc-tetrofosmin MPS patient studies were analyzed retrospectively. Studies were excluded for incomplete data, significant technical difficulties, or (for gated analysis but not for analysis of TID ratio) if the LV EF was > 75%. An analysis of gated data was undertaken to establish the reproducibility of ventricular volumes and EF between stress and rest scans. Ungated volume data were analyzed to determine the confidence limits of TID ratio according to ventricular volume.

**Results:**

Gated data were analyzed for 621 patients without inducible hypoperfusion. Mean EF at rest was slightly higher than after stress (62.4% ± 10.3% vs 61.2% ± 10.4%, *P* < 0.001), and the standard deviation of the difference was 5.2% (95% CI 4.9% to 5.5%). Ungated volumes were available for 992 non-ischaemic patients. The upper 95% CI for TID ratio was 1.23. This increased from 1.20 to 1.37 between the highest and lowest deciles of rest ungated volume.

**Conclusion:**

Using a one-day stress-rest ^99m^Tc-tetrofosmin protocol, a fall in LV EF between rest and stress of > 11.6% or a TID ratio of > 1.23 is likely to be clinically reliable. The upper limit of normal for TID ratio needs to be increased for patients with small LV chamber volumes.

**Electronic supplementary material:**

The online version of this article (10.1007/s12350-018-1253-4) contains supplementary material, which is available to authorized users.

## Introduction

Coronary artery disease (CAD) represents the leading cause of mortality and morbidity in the developed world.^1^ MPS has been recommended by the European Society of Cardiology as a first-line investigation for investigating ischemia in patients with chest pain.^2^ The extent and severity of perfusion defects on myocardial perfusion scintigraphy (MPS) provide important prognostic information.^3^ Measurements of left ventricular volume and function derived from both ungated and gated single photon emission computed tomography (SPECT) are also important predictors of risk. Thus the annual rate of cardiac death increases sharply as resting or post-stress left ventricular (LV) ejection fraction (EF) falls below 45%,^4^,^5^ or end-systolic volume increases above 70 mls.^5^

It has been demonstrated that differences in LV indices between the post-stress acquisition and the rest acquisition are clinically important. A larger LV cavity volume on the ungated SPECT images post stress compared with rest has been termed “transient ischaemic dilatation” (TID).^6^,^7^ It is uncertain whether this phenomenon represents true cavity dilatation due to post-ischaemic stunning,^8^ or generalized subendocardial hypoperfusion during stress which gives the appearance of dilatation.^9^,^10^ On occasion, TID occurs with relatively minor or even no perfusion abnormality, and can sometimes be a clue to extensive, but to some degree balanced, multi-vessel ischemia.^6^,^11^–^13^ However TID is a relatively non-specific marker of ischemia, and correlates poorly with findings on computed tomographic coronary angiography.^14^ Additionally, in those with otherwise normal MPS studies, TID does not predict CAD at coronary angiography performed within 6 months.^15^ It now appears that TID occurs almost exclusively in patients with diabetes or LV hypertrophy,^16^,^17^ and hence it may be a more important indicator of microvascular dysfunction as opposed to epicardial CAD. Regardless of its physiology, TID has repeatedly been shown to be an independent predictor of adverse outcome.^18^

Post-stress end-systolic volume, as measured by echocardiography, has been shown to be an independent predictor of mortality;^19^ and increased post-stress end-systolic volume has been suggested as a marker for ischemia. A fall in LV EF post stress compared with rest may also indicate extensive inducible ischemia and an impaired prognosis.^19^,^20^ However, the value of such measures is limited without sufficient knowledge of normal intraindividual variation between stress and rest assessments.

TID can be identified qualitatively by visual inspection of the raw data of the SPECT acquisitions and processed tomograms.^21^ Modern quantitative software can provide added confidence by fitting myocardial contours to the ungated stress and rest tomograms and calculating chamber volumes, which can be expressed as a TID ratio (stress volume/rest volume).^11^ The clinical value of this index depends on its upper limit of normal, which has been quoted as 1.23 in publications from the Cedars-Sinai team.^7^ However, this upper limit of normal is likely to be dependent on LV size, as the spatial resolution of a gamma camera is poor, and hence a relatively small error in calculated volume in a small heart will have a greater impact on TID ratio than in a larger heart. The gated stress and rest tomograms can be inspected qualitatively for stress-induced wall motion abnormalities, but recognition of a change in global LV function requires quantification. It is therefore essential to establish the normal limits of variation in LV volumes and ejection fraction derived from gated SPECT, so that the maximal allowable fall in ejection fraction post stress can be determined.

In the United Kingdom, ^99m^Tc MPS studies are frequently performed using a one-day protocol, most commonly with a stress-rest order.^22^ Typically, doses of 250/750 MBq are given.^22^ It cannot be assumed that quoted normal ranges and reproducibilities for TID ratio and gated indices obtained using higher doses apply when a low-dose acquisition has been performed.

Using a one-day stress-rest technetium-99m-tetrofosmin protocol, we investigated: (1) The variation in the upper limit of normal for TID ratio with LV size, (2) The variation in LV ejection fraction and volumes between stress and rest gated SPECT acquisitions.

## Methods

### Study Population

Patients undergoing myocardial perfusion scintigraphy between 01/01/2015 and 31/12/2015 at the John Radcliffe Hospital (Oxford, UK) were retrospectively included in the analysis. Patients > 18 year old were included if they underwent a one-day stress-rest protocol. Patients were excluded if: (1) complete data were not available preventing comparison between stress and rest volumes, and (2) if the study report indicated significant technical difficulties preventing reliable volumetric measurement. For gated analysis only, patients were excluded if the ejection fraction on either the rest or stress scan was > 75% (i.e., likely to be inaccurate and due to the poor spatial resolution of SPECT).

### MPS Protocol and Analysis

MPS was performed using a one-day stress-rest ^99m^Tc-tetrofosmin protocol. Prior to attendance, patients were asked to omit beta-blockers for 48 hours, and avoid caffeine for 12 hours. Patients were stressed using symptom-limited treadmill exercise, or where necessary regadenoson 400 mcg IV. ^99m^Tc-tetrofosmin was administered intravenously at a weight-adjusted dose of 250 to 400 MBq during stress and 750 to 1200 MBq at rest. Patients with a significant perfusion defect on the stress acquisition received sublingual glyceryl trinitrate prior to the rest injection to optimize assessment of viability/reversibility.

SPECT acquisition was performed 20 to 60 minutes after stress injections and 1 to 2 hours after rest injections using a two-headed “Pulse CDC” gamma camera (IS2) equipped with low energy general purpose tungsten foil collimators. Although termed “general purpose,” we have shown that these collimators yield a full-width at half-maximum of 8 mm at 10 cm, similar to “high resolution” collimators on most cameras. A 180° contoured orbit with 32 steps of 40 seconds each for the stress acquisition and 30 seconds for the resting acquisition was used (energy window 140 keV ± 10%, matrix 64 × 64, pixel size 6.5 mm). Acquisitions were gated whenever possible with 16 frames and an RR acceptance window of ± 40%.

Each MPS study was processed and analyzed using a Hermes workstation. Iterative (HOSEM) reconstruction was performed on the summed projections. Filtered back projection was used to reconstruct the gated projections. Tomograms were reoriented to the standard orthogonal planes of the heart. The studies were analyzed and displayed using Cedars-Sinai quantitative software (QPS and QGS), and the relevant quantitative parameters were recorded. The final clinical report was used to establish the normality of a study, or the presence of fixed or inducible perfusion defects. Data were extracted by two authors AE and ERI.

### Statistical Analysis

Continuous variables are expressed as either mean (standard deviation) or median (interquartile range [IQR]) and compared between stress and rest acquisitions using the paired Student’s *t*-test or Mann-Whitney *U*-test, respectively. Categorical and original variables are reported as frequency (percentage). To determine TIDr upper limits, participants were grouped into deciles of ungated rest volume. TIDr upper limits were calculated within each group as the mean plus 1.96 times the standard deviation. Homogeneity of variance of TIDr within each decile was assessed using Bartlett’s test. A regression model for the association between TIDr upper limits and rest ungated volume was determined using the Gauss-Newton method. Statistical analysis for this paper was performed using SAS software, Version 9.3 of the SAS system for Windows. Copyright © 2002-2010 by SAS Institute Inc., Cary, NC, USA.

## Results

**Figure 1:**
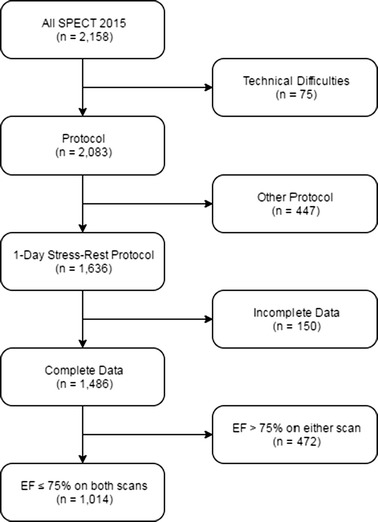
Flow chart detailing patient selection for this analysis

A total of 2158 patient studies were undertaken by the nuclear cardiology department of the John Radcliffe Hospital (Oxford, UK) during 2015. Of these, 1486 had complete volumetric data available and were suitable for inclusion in the ungated analysis. Following removal of those individuals with EF > 75%, 1014 (68.2% of studies with complete data) were suitable for inclusion in the gated analysis (Figure 1). The mean age of patients included in the analysis was 63.4 ± 12.0 and 833 (82.2%) were male, mean weight was 87.8 ± 18.1 kg (*n* = 987). Fifty-four (5.3%) had had previous CAD revascularisation, and 22 (2.2%) had passed away prior to this study (Table 1).

**Table 1:** Demographic features of participants in this cohort

Item	Complete cohort	Gated analysis	Ungated analysis
Participants	1486	621	992
Male	1014 (68.2)	495 (79.7)	624 (62.9)
Age (years)	64.4 ± 11.7	62.0 ± 12.4	63.3 ± 11.8
Weight (Kg)	85.8 ± 18.1 (*n* = 1446)	87.5 ± 17.2 (*n* = 605)	84.9 ± 17.7 (*n* = 965)
Dead prior to analysis	30 (2.0)	10 (1.6)	16 (1.6)
Stress method			
Exercise	831 (55.9)	353 (56.8)	573 (57.8)
Pharmacological	622 (41.9)	267 (43.0)	418 (42.1)
Unknown	2 (0.2)	1 (0.2)	1 (0.1)

Demographic features for the gated and ungated analysis are those participants without inducible ischemia

Those included in the gated analysis are a subset of those in the ungated analysis. Values are provided as *n*(%) or mean ± standard deviation

### Gated Analysis of Normal Limits Between Stress and Rest

Six hundred and twenty-one studies (61.2%) showed no inducible hypoperfusion (no defect 539, fixed defect only 82). In these patients, mean EF at rest was higher than after stress (62.4% ± 10.3% vs 61.2% ± 10.4%, *P* < 0.001). The mean difference was 1.2% [95% confidence interval (CI) (0.8% to 1.6%)], with a standard deviation of the difference (SDD) of 5.2 [95% CI (4.9 to 5.5)]. This change in EF was the result of a lower systolic volume (40.4 ± 26.9 mL vs 41.4 ± 28.3 mL, *P* = 0.001) and a higher diastolic volume (100.8 ± 35.7 mL vs 99.6 ± 36.3 mL, *P* = 0.002) at rest (Table 2).

**Table 2:** Volumetric data values in patients with (*n* = 389) and without (*n* = 621) inducible hypoperfusion; note four participants had inconclusive scans and are not included in these analyses

Item	Stress	Rest	Mean difference	Standard deviation of the difference	Probability
No inducible hypoperfusion (*n* = 621)					
Diastolic volume (mL)	99.6 ± 36.3	100.8 ± 35.7	1.2 (0.5 to 2.0)	9.7 (9.2 to 10.3)	0.002
Systolic volume (mL)	41.4 ± 28.3	40.4 ± 26.9	− 1.0 (− 1.5 to − 0.4)	6.9 (6.6 to 7.3)	0.001
Ejection fraction (%)	61.2 ± 10.4	62.4 ± 10.3	1.2 (0.8 to 1.6)	5.2 (4.9 to 5.5)	< 0.001
Ungated volume (mL)	71.6 ± 32.9	72.1 ± 32.0	0.4 (− 0.2 to 1.1)	8.5 (8.1 to 9.1)	0.208
Inducible hypoperfusion (*n* = 389)					
Diastolic volume (mL)	109.2 ± 37.3	107.1 ± 36.8	− 2.2 (− 3.4 to − 0.9)	12.8 (12.0 to 13.8)	0.001
Systolic volume (mL)	51.6 ± 31.6	47.9 ± 30.4	− 3.7 (− 4.7 to − 2.7)	10.0 (9.3 to 10.7)	< 0.001
Ejection fraction (%)	55.6 ± 12.2	58.1 ± 12.4	2.5 (1.8 to 3.2)	7.1 (6.6 to 7.6)	< 0.001
Ungated volume (mL)	81.4 ± 35.0	79.3 ± 34.2	− 2.1 (− 3.3 to − 0.9)	11.7 (11.0 to 12.6)	0.001

Values are mean ± standard deviation or mean (95% confidence interval). Probability refers to difference between stress and rest values

### Ungated Analysis of Normal Limits of TIDr

**Figure 2:**
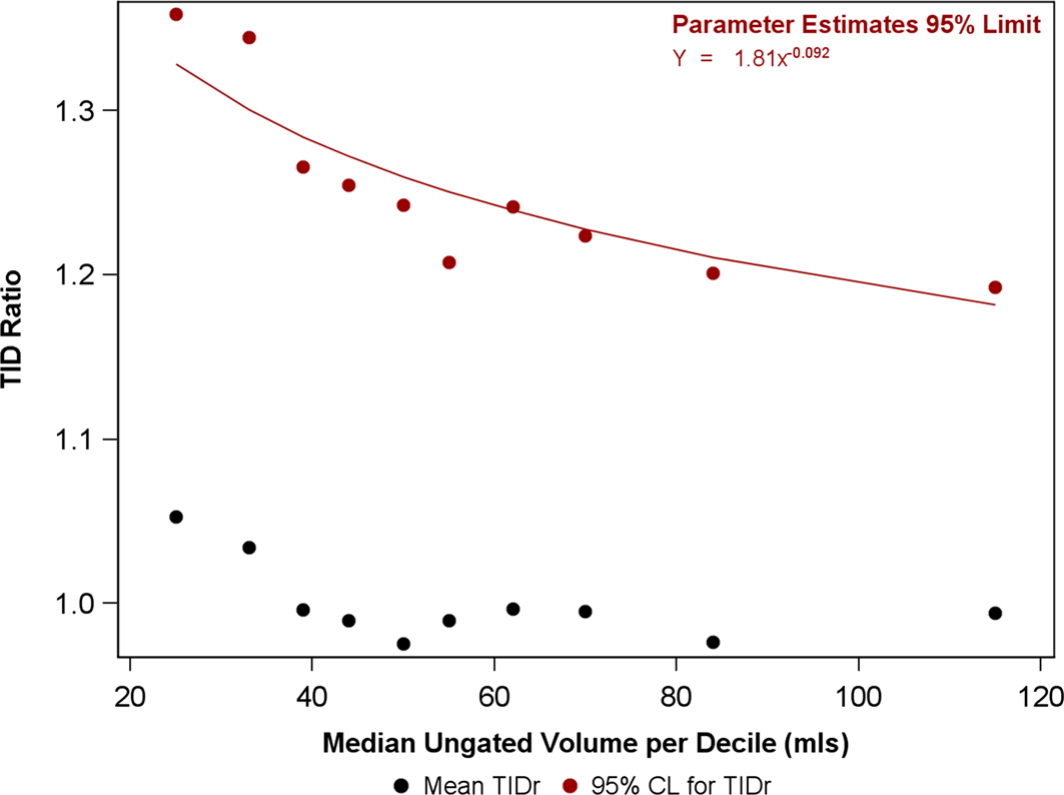
A graph displaying participants in the ungated analysis divided into deciles based on rest ungated volume. Median rest ungated volume in each decile group is plotted against mean TIDr (black) and the 95% upper confidence limit for TIDr (red). A regression line (red) is displayed for the TIDr 95% upper confidence limit and is modeled by the equation *Y* = 1.81 × *x*^−0.092^

Of those with complete data (*n* = 1486), 992 participants had no inducible hypoperfusion. In this group, the mean TID ratio (TIDr) derived from ungated volumes was 1.00 ± 0.12. These participants were divided into deciles grouped by rest ungated volumes. TIDr was associated with resting chamber volume (ANOVA *P* < 0.001). The overall upper 95% confidence limit for TIDr was 1.23. However, the upper 95% CI rose from 1.20 to 1.37 between the highest and lowest deciles of rest ungated volume (Table 2, Figure 2).

An assessment of TIDr variance between decile groups suggested heterogeneity (*P* < 0.001). Visual examination of Figure 2 suggested that the association between TIDr and resting chamber volume and TIDr heterogeneity of variance may have resulted from a significant difference in the lowest two deciles. When these two groups were removed from the analysis, significant heterogeneity of variance remained (*P* = 0.018), though TIDr was no longer associated with resting chamber volume (*P* = 0.843). The equation most closely modeling the relationship between 95% upper confidence limit of TIDr and median rest ungated volume within each decile is detailed in Table 3. Similar results for overall TID ratio and predictive equations were obtained when the cohort was split by gender (Table 4).

**Table 3:** TID ratio by rest ungated volume decile (n=992)

Rest volume decile	Participants	Median rest volume (mL)	TID ratio	
			Mean	95% Upper CL
1	99	25	1.05	1.36
2	99	33	1.03	1.34
3	99	39	1.00	1.27
4	99	44	0.99	1.25
5	99	50	0.98	1.24
6	99	55	0.99	1.21
7	99	62	1.00	1.24
8	99	70	0.99	1.22
9	99	84	0.98	1.20
10	101	115	0.99	1.19

**Table 4:** Upper 95% confidence limit for TID ratio and predictive equations in non-ischaemic participants

Cohort	N	Upper 95% CL for TIDr	Equation
All	992	1.26	${\text{TIDr}} = 1.81 \times \left( {\text{rest volume}} \right)^{ - 0.092}$
Male	631	1.24	${\text{TIDr}} = 1.80 \times \left( {\text{rest volume}} \right)^{ - 0.090}$
Female	361	1.28	${\text{TIDr}} = 2.04 \times \left( {\text{rest volume}} \right)^{ - 0.130}$

## Discussion

The results presented in this paper indicate that ejection fraction values obtained between stress and rest acquisitions demonstrate significant variability. The mean ΔEF was 1.2%, in line with previous studies.^23^ However, the standard deviation of the difference between stress and rest values was 5.2% (4.9 to 5.5). This suggests that a fall from rest to stress of at least 11.6% is required for the change to be statistically significant. The mean TIDr is 1.00, and is independent of chamber volume. However, the upper 95% confidence limit varies with chamber volume, and at smaller volumes TIDr may need to be as high as 1.37 to be significant.

This large cohort confirms the results of previous small studies (*n* < 150) which have demonstrated a 95% CL for the ΔEF of between 5.2% and 10.5%.^23^–^25^ The large number of participants in the present study allowed for selection of only those without reversible ischemia, in whom there is no physiological reason for the EF to change between the post-stress and rest acquisitions. In previous analyses the ischaemic burden was not clearly detailed,^23^,^24^ or only selected patients with defects on their stress scan were included.^25^ Ferro et al. demonstrated that a ΔEF of − 5% or more was associated with stress-induced ischemia within a cohort of patients with type-2 diabetes mellitus.^20^ However, within a non-selected population in a large cohort, our findings would suggest that such a change could be expected due to intraindividual variability and would not necessarily be clinically significant.

TID is a widely used marker that suggests the possibility of significant underlying ischemia.^26^ The upper limit for normal TIDr is dependent on the protocol and isotope used. Previous results from Xu et al. have suggested that a TIDr > 1.19 should be considered abnormal in ungated images using a one-day rest / exercise stress single isotope (^99m^Tc-sestamibi) protocol;^7^ this has been supported by a number of other groups.^27^–^30^ TIDr upper limits have been broadly similar between different software packages including QPS (TIDr 95% upper limit 1.19),^7^ Emory Cardiac Toolbox (TIDr 95% upper limit 1.19 to 1.25),^29^,^30^ and 4DM (TIDr 95% upper limit 1.24).^28^ Data from Rivero et al. support a similar overall value for TIDr but also demonstrates that this may vary by gender.^30^ Our results are in line with the findings from these groups regarding the overall TIDr (upper 95% confidence limit = 1.23) in a cohort of 992 patients with no inducible ischemia, significantly larger than other cohorts that have assessed the normal limits of TID. However when split by gender the overall TIDr remained similar, in contrast to previous studies. The large cohort size of this study allowed for stratification by chamber volume. This demonstrated that the upper 95% confidence limit for TIDr increased as chamber size decreased, suggesting that a single cut-off to define TID should be used with caution.

## Limitations

This study has a number of limitations which are acknowledged by the authors. First, it has been conducted as a retrospective analysis of ‘real world’ clinical data. The LV indices were not remeasured by the trial team. Although key findings have been established in patients without inducible ischemia on SPECT, the underlying population is heterogeneous and represents all-comers referred for investigation in the nuclear cardiology department of a tertiary center. The above limitations may contribute to an increase in the variability seen between patients; however, the intraindividual variability should be minimally impacted.

This study was undertaken using Cedars-Sinai QPS and QGS software. Reports of TIDr 95% upper limits using different software packages have been generally comparable. However, the findings of this study should be considered in the knowledge that they represent results from a single center using a single ^99m^Tc MPS protocol and analyzed by a single software package. Additional analyses by other groups is required to determine more generalizable applicability.

This study removed those with an LVEF > 75% from inclusion in the gated analysis. This was to remove those with non-physiological ejection fractions which are likely to be numerically inaccurate, and a reflection of the poor spatial resolution of a gamma camera system in small hearts. This led to the removal of 31.8% of scans with complete data from the gated analysis, and disproportionately affected women (Table 1). Such patients are generally regarded as having “normal” LV systolic function, but we do not quote the LVEF values in clinical practice. Previous studies in this area have either included patients with LVEF > 75% uncritically,^23^,^24^ or have appeared not to include patients with such values (perhaps because they were excluded from the outset).^25^ We would argue that inclusion of such data would distort the results of the gated analysis.

## Conclusion

In a single center analysis, this study represents the largest cohort to assess variability in volumetric measures and TIDr using a one-day stress-rest ^99m^Tc MPS protocol. An analysis of gated volumetric data shows a small but significant variation in EDV, ESV, and LVEF between stress and rest acquisitions. Although a decrease in LV EF between rest and post-stress acquisitions may be suggestive of ischemia, this needs to exceed 11.6% before it can be considered outside of normal variation. TIDr values assessed using non-gated volumes show an upper confidence limit of 1.23 overall. However, there is significant variation with rest ungated volume which should be taken into consideration when determining clinical significance.

## New Knowledge Gained

1. Using a one-day stress rest ^99m^Tc MPS protocol, a fall in LV EF from rest to post stress of > 11.6% is required for the change to be statistically significant (and hence clinically reliable).
2. Overall, a TIDr of > 1.23 is likely to be statistically reliable, but for small chamber volumes (< 50mLs) higher cut-offs are appropriate.

## Electronic supplementary material

Below is the link to the electronic supplementary material.

Supplementary material 1 (PPTX 198 kb)

## Abbreviations

CAD: Coronary artery disease
CI: Confidence interval
EF: Ejection fraction
LV: Left ventricle
MPS: Myocardial perfusion scintigraphy
SDD: Standard deviation of the difference
SPECT: Single photon emission computed tomography
TID: Transient ischaemic dilatation
TIDr: Transient ischaemic dilatation ratio
