# Supplementary material for: How variable are the volumetric measurements from gated perfusion SPECT when a one-day stress-rest protocol is used?
Source: J Nucl Cardiol. 2018 Mar 15;26(5):1709–16. doi: 10.1007/s12350-018-1253-4 (PMC6775029; doi:10.1007/s12350-018-1253-4)
Supplement: Supplementary file 1 — Supplementary material 1 (PPTX 198 kb) [file 12350_2018_1253_MOESM1_ESM.pptx]

## Slide 1
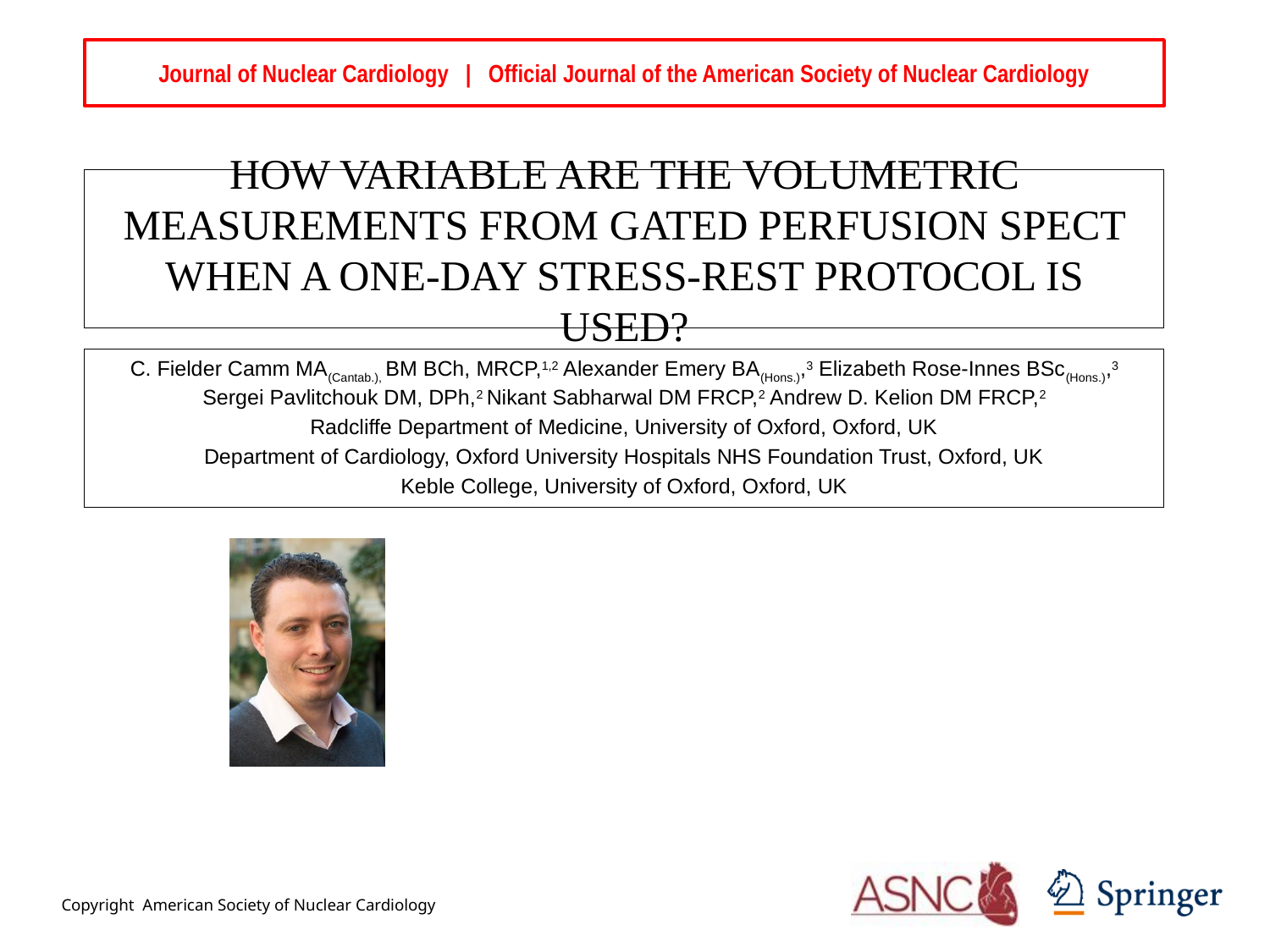

Journal of Nuclear Cardiology | Official Journal of the American Society of Nuclear Cardiology
# How variable are the volumetric measurements from gated perfusion SPECT when a one-day stress-rest protocol is used?
C. Fielder Camm MA(Cantab.), BM BCh, MRCP,1,2 Alexander Emery BA(Hons.),3 Elizabeth Rose-Innes BSc(Hons.),3 Sergei Pavlitchouk DM, DPh,2 Nikant Sabharwal DM FRCP,2 Andrew D. Kelion DM FRCP,2
Radcliffe Department of Medicine, University of Oxford, Oxford, UK
Department of Cardiology, Oxford University Hospitals NHS Foundation Trust, Oxford, UK
Keble College, University of Oxford, Oxford, UK
Copyright American Society of Nuclear Cardiology

## Slide 2
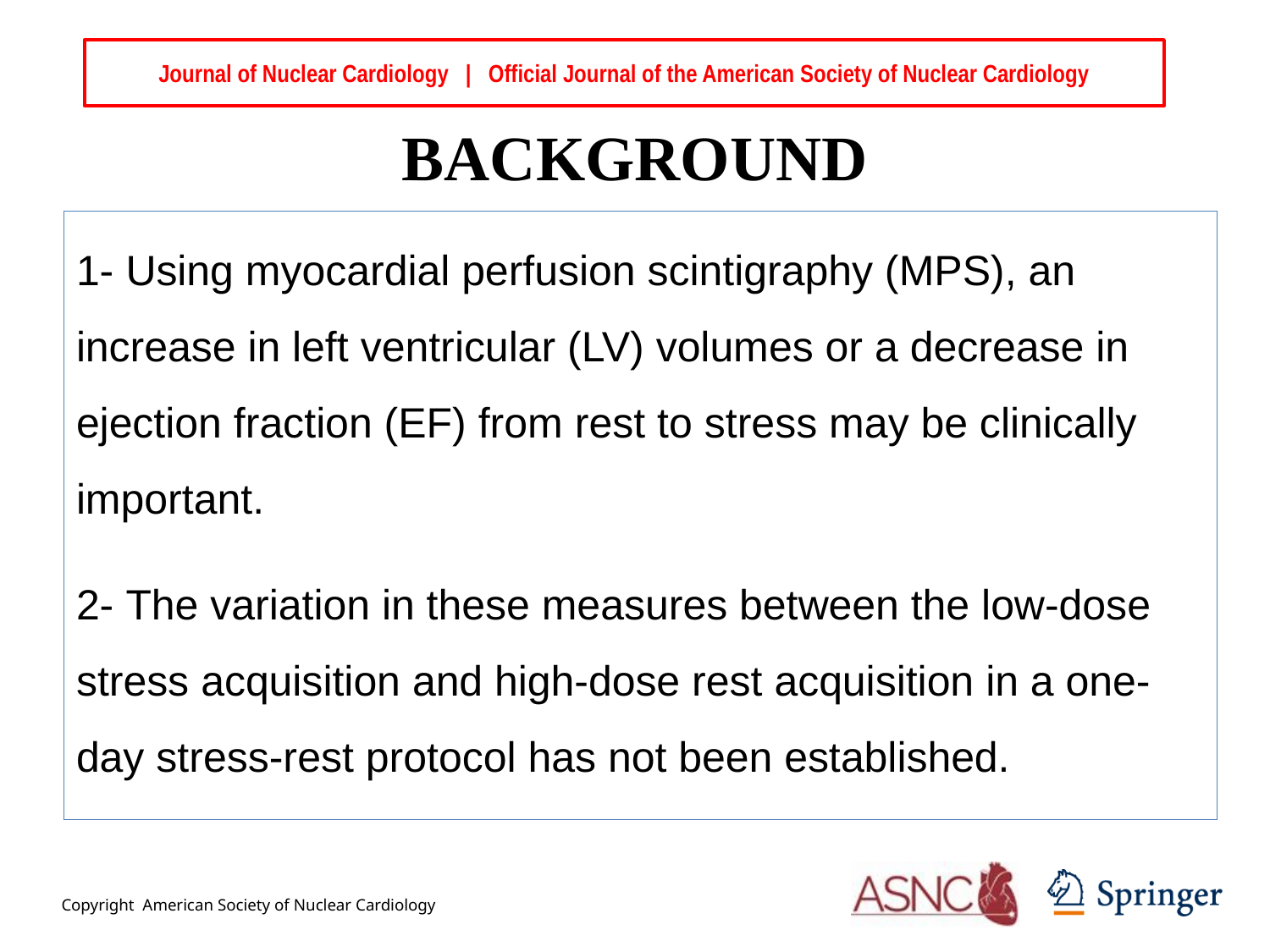

Journal of Nuclear Cardiology | Official Journal of the American Society of Nuclear Cardiology
# BACKGROUND
1- Using myocardial perfusion scintigraphy (MPS), an increase in left ventricular (LV) volumes or a decrease in ejection fraction (EF) from rest to stress may be clinically important.
2- The variation in these measures between the low-dose stress acquisition and high-dose rest acquisition in a one-day stress-rest protocol has not been established.
Copyright American Society of Nuclear Cardiology

## Slide 3
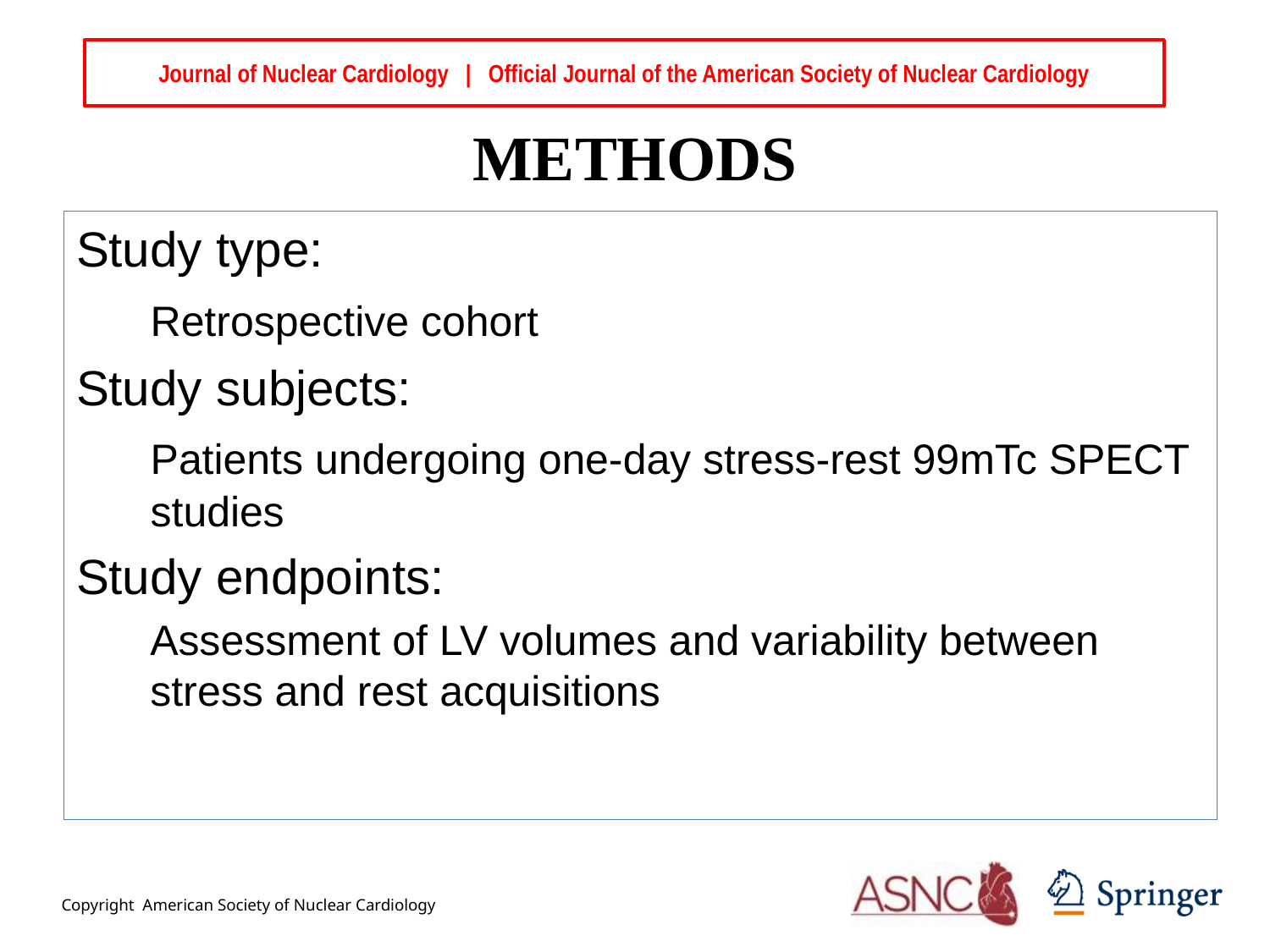

Journal of Nuclear Cardiology | Official Journal of the American Society of Nuclear Cardiology
# METHODS
Study type:
	Retrospective cohort
Study subjects:
	Patients undergoing one-day stress-rest 99mTc SPECT studies
Study endpoints:
Assessment of LV volumes and variability between stress and rest acquisitions
Copyright American Society of Nuclear Cardiology

## Slide 4
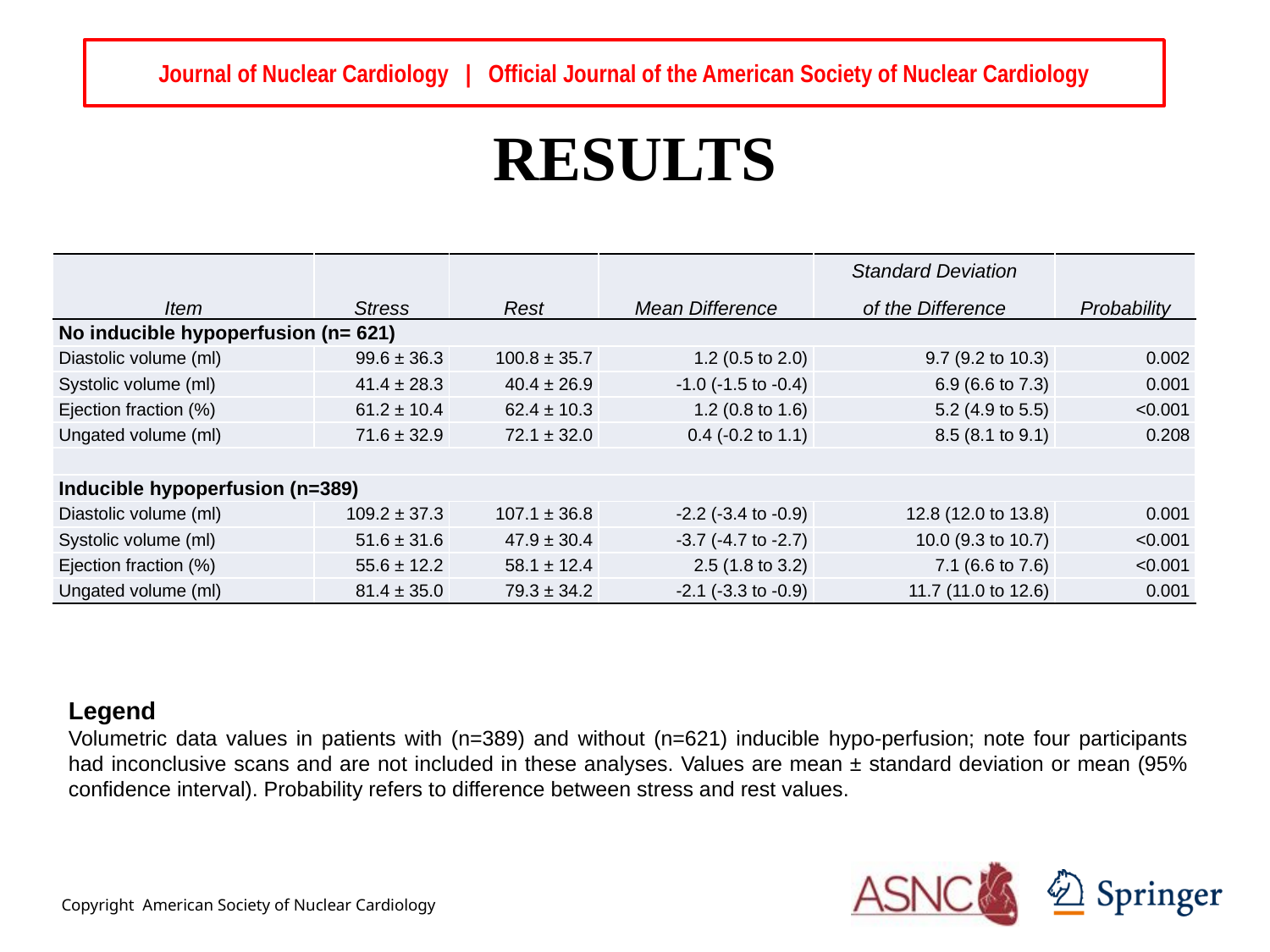

Journal of Nuclear Cardiology | Official Journal of the American Society of Nuclear Cardiology
# RESULTS
| Item | Stress | Rest | Mean Difference | Standard Deviation of the Difference | Probability |
| --- | --- | --- | --- | --- | --- |
| No inducible hypoperfusion (n= 621) | | | | | |
| Diastolic volume (ml) | 99.6 ± 36.3 | 100.8 ± 35.7 | 1.2 (0.5 to 2.0) | 9.7 (9.2 to 10.3) | 0.002 |
| Systolic volume (ml) | 41.4 ± 28.3 | 40.4 ± 26.9 | -1.0 (-1.5 to -0.4) | 6.9 (6.6 to 7.3) | 0.001 |
| Ejection fraction (%) | 61.2 ± 10.4 | 62.4 ± 10.3 | 1.2 (0.8 to 1.6) | 5.2 (4.9 to 5.5) | <0.001 |
| Ungated volume (ml) | 71.6 ± 32.9 | 72.1 ± 32.0 | 0.4 (-0.2 to 1.1) | 8.5 (8.1 to 9.1) | 0.208 |
| | | | | | |
| Inducible hypoperfusion (n=389) | | | | | |
| Diastolic volume (ml) | 109.2 ± 37.3 | 107.1 ± 36.8 | -2.2 (-3.4 to -0.9) | 12.8 (12.0 to 13.8) | 0.001 |
| Systolic volume (ml) | 51.6 ± 31.6 | 47.9 ± 30.4 | -3.7 (-4.7 to -2.7) | 10.0 (9.3 to 10.7) | <0.001 |
| Ejection fraction (%) | 55.6 ± 12.2 | 58.1 ± 12.4 | 2.5 (1.8 to 3.2) | 7.1 (6.6 to 7.6) | <0.001 |
| Ungated volume (ml) | 81.4 ± 35.0 | 79.3 ± 34.2 | -2.1 (-3.3 to -0.9) | 11.7 (11.0 to 12.6) | 0.001 |
Legend
Volumetric data values in patients with (n=389) and without (n=621) inducible hypo-perfusion; note four participants had inconclusive scans and are not included in these analyses. Values are mean ± standard deviation or mean (95% confidence interval). Probability refers to difference between stress and rest values.
Copyright American Society of Nuclear Cardiology

## Slide 5
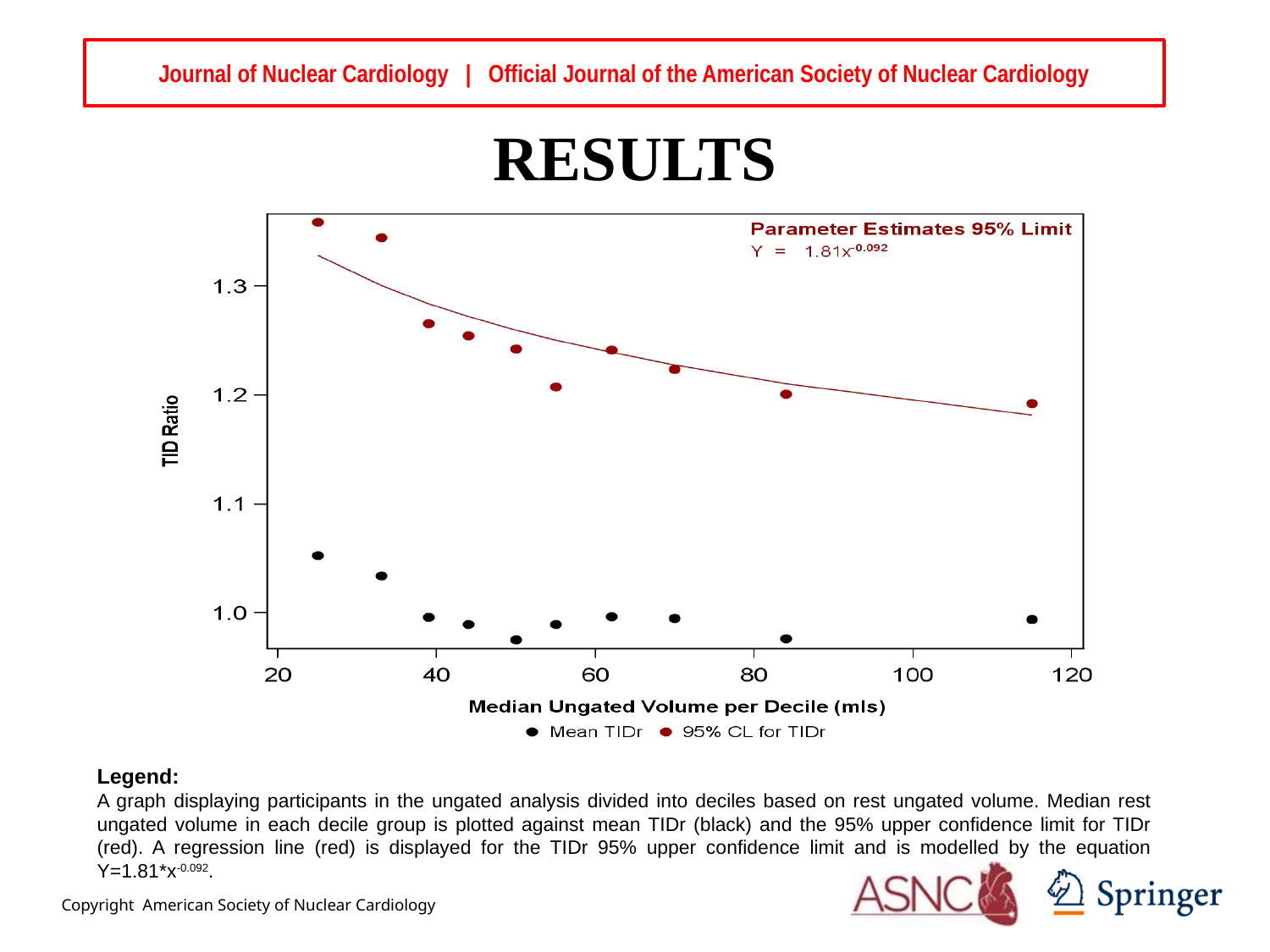

Journal of Nuclear Cardiology | Official Journal of the American Society of Nuclear Cardiology
# RESULTS
Legend:
A graph displaying participants in the ungated analysis divided into deciles based on rest ungated volume. Median rest ungated volume in each decile group is plotted against mean TIDr (black) and the 95% upper confidence limit for TIDr (red). A regression line (red) is displayed for the TIDr 95% upper confidence limit and is modelled by the equation Y=1.81*x-0.092.
Copyright American Society of Nuclear Cardiology

## Slide 6
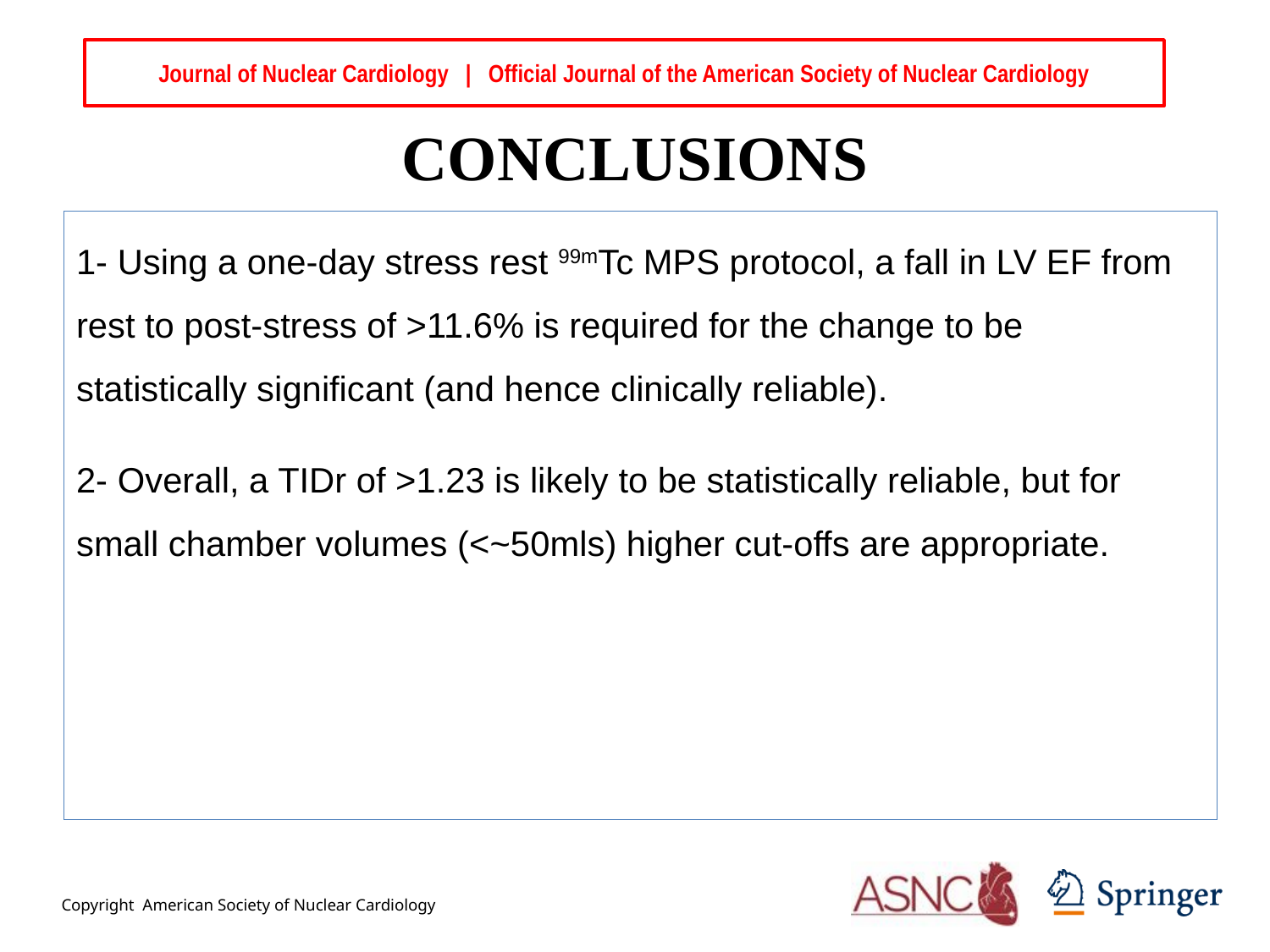

Journal of Nuclear Cardiology | Official Journal of the American Society of Nuclear Cardiology
# CONCLUSIONS
1- Using a one-day stress rest 99mTc MPS protocol, a fall in LV EF from rest to post-stress of >11.6% is required for the change to be statistically significant (and hence clinically reliable).
2- Overall, a TIDr of >1.23 is likely to be statistically reliable, but for small chamber volumes (<~50mls) higher cut-offs are appropriate.
Copyright American Society of Nuclear Cardiology
